# E-cigarette Blast Injury: Complex Facial Fractures and Pneumocephalus

**DOI:** 10.5811/westjem.2016.7.31354

**Published:** 2016-10-20

**Authors:** Benjamin A. Archambeau, Stephanie Young, Carol Lee, Troy Pennington, Christopher Vanderbeek, Dan Miulli, John Culhane, Michael Neeki

**Affiliations:** * Arrowhead Regional Medical Center, Department of Emergency Medicine, Colton, California; † Arrowhead Regional Medical Center, Department of General Surgery, Colton, California; ‡ Arrowhead Regional Medical Center, Department of Neurosurgery, Colton, California Loma Linda University Medical Center, Department of Oral Maxillofacial Surgery; § Loma Linda, California

## Abstract

Electronic cigarettes (also known as e-cigarettes or e-cigs) are becoming a popular method of recreational nicotine use over recent years. The growth of new brands and devices has been outpacing the FDA’s ability to regulate them. As a result, some of these devices fail without warning, most likely from malfunction of the lithium-ion batteries that are in close proximity to volatile compounds within the device. Failures have occurred during both use and storage of the devices or their components. The subsequent injuries from several of these events, including full thickness burns requiring grafting and blast injuries, have been observed at Arrowhead Regional Medical Center, a regional trauma and burn center in southern California. One severe case resulted in several maxillofacial fractures, blurred vision, and pneumocephalus after a device failed catastrophically during use. The patient required close monitoring with serial imaging by neurosurgery in the intensive care unit and multiple procedures by oral maxillofacial surgery to reconstruct his facial bones and soft tissue. Ultimately, the patient recovered with minimal permanent damage, but the potential for further injury or even death was apparent. Cases such as this one are becoming more frequent. It is important to increase awareness of this growing problem for both medical professionals and the general public in order to curb this concerning new trend.

## INTRODUCTION

The Federal Emergency Management Agency (FEMA) reported in 2014 that there were over 2.5 million e-cigarette users in the United States, and since then that number has grown substantially.^1^ The introduction of electronic cigarettes (ECs) and the rapid growth in both use and manufacturing of such products in recent years has prompted the need for more research, regulation, and awareness about the potential hazards of these devices and their contents. These devices use a battery-powered heating filament to aerosolize volatile compounds, such as propylene glycol or glycerin, nicotine, and flavoring compounds.

Patients are presenting to emergency departments (EDs) with not just thermal but also severe blast injuries that have occurred during both the use and storage of ECs. To date, only limited studies exist on the safety of the inhaled substances, secondhand exposure, and the devices themselves. At least 18 cases of EC explosions have presented to Arrowhead Regional Medical Center (ARMC) in Colton, California, a regional trauma and burn center serving San Bernardino and several surrounding counties. Injuries range from mild first and second degree superficial burns to complex craniofacial fractures requiring intensive medical and surgical care. Here we present a case of explosive failure of an EC resulting in severe maxillofacial and skull fractures leading to pneumocephalus.

## CASE REPORT

A 59-year-old male with history of leukemia, hyperlipidemia, chronic back pain and baseline right hearing loss was airlifted to ARMC for trauma evaluation following the explosion of an e-cigarette while “vaping.” The patient had received the device two days prior after purchasing it online and reportedly made no modifications.

**Figure 1:**
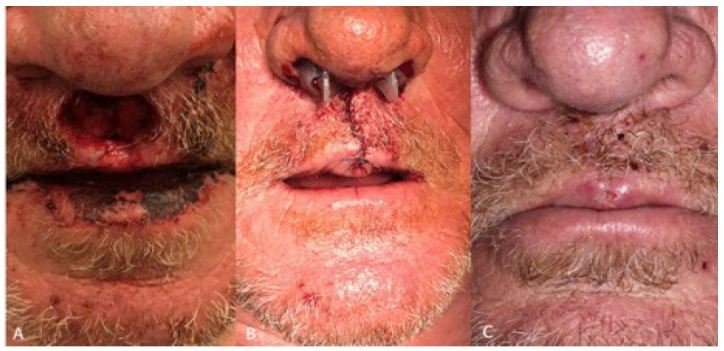
**A.** excisional blast injury to philtrum. **B.** Philtrum and nares after surgical repair and packing by oral maxillofacial surgery. **C.** Well healed wound at follow-up visit.

**Figure 2:**
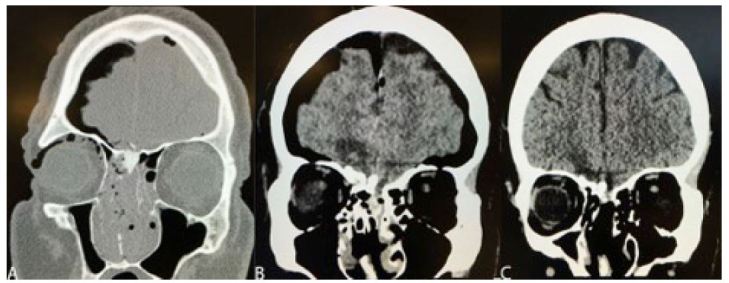
**A.** Maxillofacial computed tomography from outside facility demonstrating ethmoid, nasal, and cribriform fractures with pneumocephalus, with **B.** progression over the initial 24 hours following injury, and **C.** resolution of pneumocephalus at one month.

On arrival to the ED, the patient was alert, oriented, and calm with an initial Glasgow Coma Scale of 15. He complained of epistaxis, facial pain, blurry vision in his right eye, and decreased hearing in the left ear. His physical exam was significant for palpebral edema and ecchymosis of the right eye, maxillary tenderness and gross blood in the oropharynx without brisk bleeding, and a circular avulsion injury to the philtrum (Figure 1). In addition, he was covered with soot on his lips, right hand and the right side of his face. Computed tomography (CT) showed fractures of the petrous, ethmoid, cribriform plate, nasal choanae, nasal septum and right medial orbital wall as well as pneumocephalus (Figure 2). He also sustained a right periorbital contusion. A nearly completely avulsed philtrum communicated intraorally and through the nasal mucosa bilaterally with exposure of nasal septal cartilage.

Oral and maxillofacial surgery (OMFS) performed reduction and splinting of the nasal fractures and repair of the philtrum and nasal floor defects. The patient was admitted to the surgical intensive care unit for close monitoring of neurologic function. Neurosurgery recommended a repeat CT head to re-evaluate pneumocephalus the following day, and it was found to have increased in size. The patient was continued on supplemental oxygen via non-rebreather mask to aid in resorption of pneumocephalus, which has been demonstrated as effective management in patients with pneumocephalus following craniotomy.^2^ A repeat CT head two days later showed that the pneumocephalus had improved. Ophthalmological evaluation of the right eye blurriness and periorbital contusion did not reveal any significant eye injury and daily artificial tears were recommended. The patient reported clinical improvement of blurry vision and diminished hearing in the left ear prior to his discharge. He had no change in neurologic function and was discharged home on hospital day three with instructions to follow up with neurosurgery within two weeks and with OMFS within one week.

The patient followed up with OMFS one week after the incident, at which time the nasal splints and lip sutures were removed without complication (Figure 1). At a one-month follow-up visit with the ARMC neurosurgery department, a repeat CT of the brain demonstrated the complete resolution of the pneumocephalus (Figure 2) and his facial injuries were well healed with minimal residual scarring. He had no neurological deficits at that time. The only potential complication was persistent yellow-green nasal discharge, possibly indicative of a sinus infection.

## DISCUSSION

It is currently thought that EC explosions are caused by the proximity of the heating element to an improperly insulated lithium ion battery and its exposure to volatile liquids. Reported cases of possible EC failures are consistent with lithium ion battery failures observed in other devices. The proposed mechanism of failure has been well documented.^1^ The failure is potentially more frequent and more harmful in ECs, due to their configuration and proximity to users’ faces. This high rate of failure may be caused primarily by design flaws or manufacturing defects and exacerbated by user device modification, or even due to common storage situations. In this case, the patient was using the device, but other cases have been noted of spontaneous failure during storage and transport. Cases of documented blast injury have demonstrated directionality toward the upper and posterior oral cavity and palate causing fractures, burns, lacerations and dental injuries, including avulsion and fracturing of the teeth.^3^,^4^ These injuries have the potential to be permanently disfiguring and disabling with serious neurologic sequelae.

Management of injuries sustained from EC explosions should be approached from the standpoint of addressing both thermal and chemical burns, as well as concussive blast injuries. Patient clothing should be removed as there may be residual nicotine or other chemicals present. There should be a high suspicion for occult, possibly severe maxillofacial and cranial injuries. Providers should also consider pulmonary irritation from inhalants and potential overdoses of nicotine or other substances in any patient exposed to open cartridges; there is potential for acute lung injury in these cases.^5^

## CONCLUSION

The cases observed at ARMC over the last several months are only a small portion of potential problems in an entirely new field of the consumer market, but are likely to present daily in EDs across the country. The EC products market is booming in America. With their growing popularity, these products are being produced and distributed at a rate that exceeds the FDA’s current ability to monitor, test, and regulate them. They are sold widely on the Internet, making them more difficult to track. These devices are also modifiable beyond the manufacturer’s recommendations. This is particularly dangerous as these modifiable parts include a heating element in close proximity to lithium ion batteries, which have highly exothermic and potentially explosive consequences under certain circumstances. Compatibility issues between brands and replacement parts may also lead to device failures, which can cause catastrophic injuries.

The alarming severity and increasing frequency of catastrophic failures warrants further investigation into the safety of ECs. Whether failure is caused by the device as a whole, the battery, or any of its other components, the injuries that have resulted are well out of reasonable tolerances. Even if most or all of the accidents are due to user modification or improper storage, there needs to be further safety mechanisms in place. In the meantime, focus should be on increasing public awareness along with hospital and ED provider education across the country to treat the victims.
